# Supplementary material for: A Global Screen for Assembly State Changes of the Mitotic Proteome by SEC-SWATH-MS
Source: Cell Syst. 2020 Feb 26;10(2):133–155.e6. doi: 10.1016/j.cels.2020.01.001 (PMC7042714; doi:10.1016/j.cels.2020.01.001)

Q10567 | AP1B1_HUMAN | AP1B1 ADTB1 BAM22 CLAPB2
Monomer MW [kDa]: 104.637 Monomer expected elution fraction: 40

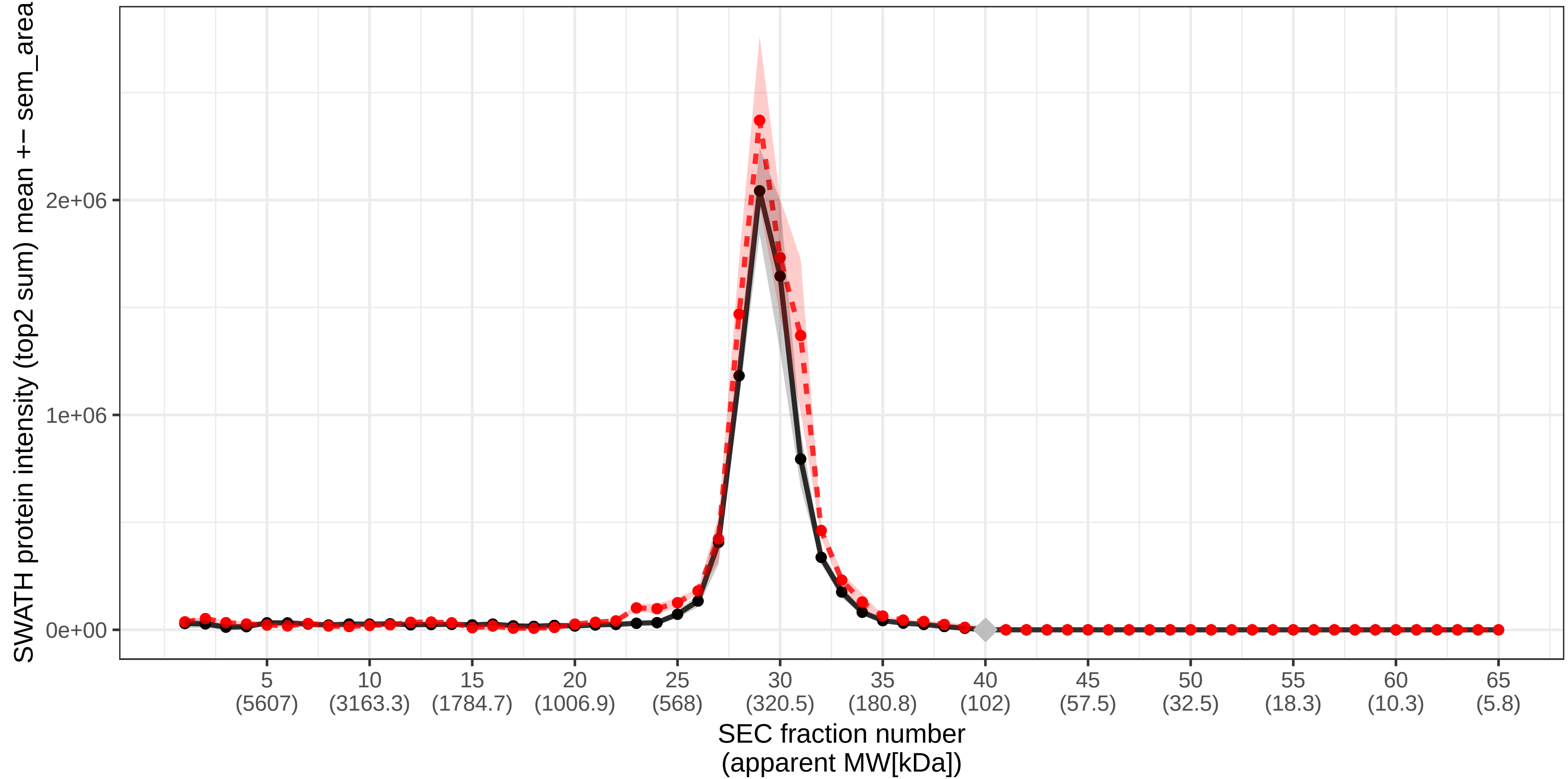

Supplement: Data S1. SEC-SWATH-MS Protein Chromatograms, Related to Figure 1 [file mmc6.zip › SECchrom_Q10567_AP1B1_HUMAN_AP1B1_ADTB1_BAM22_CLAPB2.pdf]
